# A commentary on studies presenting projections of the future prevalence of dementia

**DOI:** 10.1186/1471-2458-13-1

**Published:** 2013-01-02

**Authors:** Sam Norton, Fiona E Matthews, Carol Brayne

**Affiliations:** 1 Institute of Public Health, University of Cambridge, Robinson WayCambridge, CB2 0SR, UK; 2 MRC Biostatistics Unit, Robinson Way, Cambridge, CB2 0SR, UK

## Abstract

**Background:**

Population ageing over the first half of this century is likely to lead to dramatic increases in the prevalence of dementia. This will affect all regions of the world, but particularly developing regions. Dementia projections have been used extensively to support policy. It is therefore important these projections are as accurate as possible.

**Discussion:**

In this paper we provide a commentary on studies projecting the future prevalence of dementia for the world or for individual continents. We identify some important limitations of the methods used in published projections and provide recommendations to improve the accuracy of future projections, and allow for the checking of the accuracy of the predictions.

**Summary:**

Accurate projections of dementia incidence, at both the global and local level, are essential for healthcare planners.

## Background

The prevalence of dementia is a major concern for future global public health [1]. Population ageing, driven by greater life-expectancy and low fertility, will result in a dramatic growth in the older population in all regions. By the middle of this century around 1 in 5 of the estimated 9 billion world population are expected to be aged over 60-years, compared to around 1 in 10 in 2000 [2]. Since dementia incidence increases exponentially with age [3], the likely future number of dementia cases and associated burden on the healthcare system is of considerable concern to healthcare planners [4,5].

**Figure 1:**
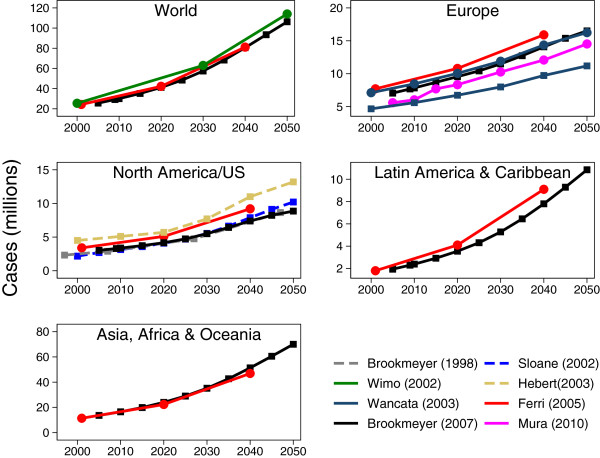
**Dementia projections.** Lines with circle markers indicate dementia; lines with square markers indicate Alzheimer’s disease; dashed lines indicate projection is for US only. The figure shows projections from previously published projections by continent.

So that our evaluation is evidence-based we conducted a scoping exercise to identify studies, published in peer reviewed journals, presenting projections concerning the world as a whole or for individual continents. (Details of these studies, along with the search strategy and selection criteria, are given in the online Additional file 1) This literature search identified a large and growing number of dementia projections. Unequivocally, the projections indicated that, irrespective of region, the projected number of cases of dementia is predicted to rise dramatically during the first half of this century (Figure 1). Furthermore, due to the larger expected gains in life expectancy for developing countries, steeper increases in the estimated number of cases of dementia in the regions consisting of Latin America and the Caribbean, and Africa, Asia and Oceania compared to Europe and North America [6,7].

These estimates provide a gloomy outlook. However, it is not clear to what extent these projections compare with current prevalence estimates. For example, although projections suggest a dramatic increase in dementia, there is evidence that the prevalence over the past 20 years has remained stable or even reduced [8-10]. Dementia projections make several important assumptions about the stability of dementia prevalence and incidence over time and across regions that are likely to impact on accuracy of the projection. These assumptions further suppose that dementia risk factors will also remain stable, which is highly unlikely. Projections need to account for likely changes in the future prevalence of dementia risk factors, as well as uncertainty regarding their true causal link with dementia. In this paper we describe the limitations of the methods used by the dementia projection studies and offer recommendations that will help to increase the validity and reliability of future projections.

## Discussion

The dementia projections identified used two different methodologies (see Additional file 1): extrapolations [7,11,12] and macro-simulations [3,13-15]. Extrapolations estimate the future number of cases of dementia by simply multiplying the expected number people within specific age (and sometimes sex) strata from an existing population projection by an estimate of the current prevalence of dementia within the same strata. Providing a simplistic example, the UN estimates that there will be 1 billion people aged 65 and over in 2050 [2], if we assume the current prevalence in over 65’s is 7% then we would extrapolate the number of cases of dementia in the over 65’s in 2050 to be 70 million (1,000,000,000×.07 = 70,000,000). Macro-simulations apply a slightly different approach. Current population estimates stratified by age (and possibly other factors, such as sex) are used as the baseline population, and the current number of dementia cases calculated using existing prevalence estimates. Then, using transition probabilities from a multistate illness-death model, the number of incident cases of dementia and deaths within a specified time interval is calculated. The model is then aged forward by the specified time interval, accounting for the transitions to dementia or death, and updated using numbers for the youngest age group from existing population projections. The number of incident cases of dementia and deaths within the next interval is then calculated. This is run iteratively up to the end year.

The assumptions underlying these approaches are summarised in Table 1 and the main advantages and disadvantages of the methods along with a third method, micro simulation, is presented in Table 2. The limiting impact of the assumptions underlying extrapolations and macro simulations on projection accuracy are now discussed.

**Table 1:** Projection method assumptions

**Extrapolation**	**Cell-based macro-simulation**
Constant prevalence over time	Constant incidence over time
Constant prevalence between regions	Constant incidence between
	regions
Equal mortality risk	Constant difference in mortality
	risk
Constant rate of progression	Rate of progression stable over
	time
Risk factors stable over time	Risk factors stable over time
Risk factors stable between regions	Risk factors stable between
	regions

**Table 2:** Advantages and disadvantages of projection methods

	**Extrapolation**	**Macro simulation**	**Micro simulation**
Complexity	Simple	Not overly complex	Complex
Account for excess mortality in dementia	No	Yes	Yes
Account for changes in incidence or prevalence	Yes	Yes	Yes
Account for changes in progression	No	Yes	Yes
Directly account for changes in risk factors	No	No	Yes
Allows identification of distributional characteristics of systems	No	No	Yes

### Mortality differential

The extrapolation method makes several strong assumptions. Perhaps the least feasible is that mortality rates are assumed to be equal in healthy and demented groups. A considerable body of research has highlighted the increased mortality associated with dementia [16]. Failure to account for this is likely to upwardly bias projections. Cell-based macro-simulations that assume transition from a healthy state to dementia and from the healthy and demented states to death explicitly account for the mortality differential. Some studies additionally include transition from early to late-stage dementia [6,15], accounting for the increased mortality risk in more severe disease.

### Stability of risk factors

Both extrapolations and multi-state models make strong assumptions concerning the constancy of certain factors over time. For extrapolations, the assumption is that the relative prevalence does not change with time, other than as a result of changes in demographic factors that are used as strata in the calculations. In contrast, inputs to multi-state models (e.g. incidence, progression and mortality), although typically assumed to be constant over time in the baseline model, can be modified to reflect different future scenarios. However, other factors that are not inputs to the models are implicitly assumed constant over time, such as the prevalence of risk factors. It is important to note that the aim of a projection is not necessarily to forecast the future but to highlight the extent of the problem if risk factor trends remain stable. However, examining the impact of changing patterns of risk factors is key since it identifies targets most likely to lead to effective intervention.

Several studies employing cell-based macro-simulations examined hypothetical scenarios of delayed incidence or progression of dementia [3,15,17]. The relatively crude analyses conducted assume a step change in rates of incidence or progression at a specific time. Miracle cures are often heralded following scientific breakthroughs in our understanding of pathology but seldom are realised. In actuality changes in rates of incidence and progression of dementia are more likely to come from a combination of factors, with substantial contribution from risk factor modification [18,19]. For example, a recent study suggested population attributable risk relating to modifiable risk factors — such as diabetes, mid-life hypertension, obesity, smoking, physical or cognitive inactivity and depression — to be in the order of 50% [18]. Although this estimate is likely to be considerably inflated due to the assumption of independence of risk factors in its calculation, the contribution of behavioural factors to both the incidence and progression of dementia is clearly not insignificant. This raises the issue that modification of behavioural risk factors may impact on incidence and progression gradually and over an extended period of time. This issue is ignored by all current dementia projections. Furthermore, it is unclear whether changes in the treatment of other conditions may impact on the prevalence of dementia. For example, increased post-stroke survival may lead to increases in dementia incidence [20,21] but there is also some evidence suggesting statins may have some preventative implications for dementia [22]. It is clear that the accuracy of projections that do not address these issues is limited.

A related issue is the timing of the presence of risk factors. Treves and Korczyn highlight the importance of considering the age at which exposure to risk factors begins since their effect “may depend upon the age at which they act and their interaction with other factors or concomitant conditions, which underlines the importance of stratified and multivariate analyses by period of exposure” [23]. For example, mid-life diabetes and hypertension are more predictive of development of AD than the presence of these conditions in later periods [18]. There is only limited utility in modelling the impact of late-life hypertension if mid-life hypertension is a more important predictor.

Future analyses must be based on the assessment of more plausible scenarios regarding changes in patterns of risk factors, and their influence on both the incidence of dementia and its progression over time along with the examination of life-course influences through age, period and cohort effects.

### Generalisibility

Assumptions of constancy also apply across countries. Risk factor profiles are likely to differ across countries at the present time but are also likely to follow different future trends and therefore generalising estimates of prevalence and incidence assuming constancy over time and over countries may not be the best possible approach. This is emphasised by the studies projecting future prevalence of dementia in Latin American, African and Asian populations [6,7]. The estimates from Brookmeyer et al. were solely based on the pooled incidence estimates of US studies, which tend to be higher than for other regions [24]. The resultant projections for Asia, Africa and Oceania with respect to AD were higher than those for all dementias projected by Ferri et al. who allowed prevalence to vary across countries using a Delphi consensus study. The Delphi method attempts to account for demographic differences between regions by developing region specific age and sex stratified prevalence estimates guided by expert panel consensus. Nevertheless, the Delphi method still fails to systematically account for likely future changes in the demographic structure that may have a bearing on future dementia prevalence.

A further factor impacting on generalisibility is that studies publishing projections typically rely on data concerning the prevalence and incidence drawn from a range of sources. For example, Brookmeyer et al. [3] pooled prevalence rates across four US studies, whereas Wancata et al. [11] pooled estimates from multiple meta-analyses. The use of summary data has the advantage that it may more closely generalise to the population of interest — as long as estimates are pooled across homogenous samples. Individual data however allow for the building of more complex models that can account more appropriately for changes in underlying risk factors [25].

### Recommendations for future projections

Recent developments in multistate modelling allow for the extension to the illness-death models employed in macro-simulation projections to more appropriately account for issues such as the timing of dementia onset and potential misdiagnosis [26]. Currently illness-death models employed in macro-simulation studies use a discrete-time framework. Using a continuous-time framework allows for a more fine grained examination of the changing hazard for development of dementia or death over time that will lead to more accurate estimates of dementia incidence in light of underlying risk factors. Another development is the ability to handle potential misclassification within the model thus accounting for potential misdiagnosis of dementia [27].

Micro-simulation also provides an opportunity to further develop existing dementia projection models (Table 2). The use of micro-simulation allows for dynamic modelling of ageing and health estimation enabling more robust conclusions to be drawn with regard to the influence of changing patterns of underlying risk factors and the impact of policy changes. For example, Canadas Population Health Model (POHEM) uses individual level data concerning socio-demographic, behavioural and biomedical factors to simulate morbidity and mortality for various diseases and is used to evaluate competing health care scenarios for specific diseases [28]. A disadvantage of micro-simulation is that the method can be rather complicated to implement.

## Summary

In conclusion, population ageing is likely to lead to dramatic increases in the prevalence of dementia in future years. Providing accurate estimates of the future number of cases of dementia is essential for healthcare planners. Current estimates often extrapolate age-specific prevalence estimates to existing population projections or use macro-simulations based on age-specific dementia incidence. While such projections are useful in highlighting the scale of the problem were risk factors to remain stable over time, this is clearly an untenable assumption. The development of sophisticated projection models that not only account for the incidence, progression and excess-mortality of dementia but also allow for detailed examination of the influence of changing patterns of risk factors on future prevalence is essential. This will enable researchers to target modifiable risk factors identified by epidemiological studies where intervention is most likely to delay dementia onset, thus reducing overall prevalence in future populations. Complex interrelationships and uncertainties about the causal status of dementia risk factors pose a problem for multi-state models. However, such models allow scenarios where changes in the prevalence and incidence of risk factors, or the strength of their causal association with dementia, may be compared.

We do not suggest the outright dismissal of projections based on extrapolations and macro-simulation methods but recommend an increased investment in micro-simulation as a complement to existing projections. Applying estimates from a single source that is appropriate to the population of interest using both a macro and micro-simulation framework will allow for detailed examination of uncertainty around the projections and the impact of changes in underlying patterns of risk factors.

## Abbreviations

AD: Alzheimer’s disease; CFAS: Cognitive Function and Ageing Studies.

## Competing interests

The authors declare that they have no competing interests.

## Authors’ contributions

SN conducted the literature search and drafted the manuscript. CB and FM conceived of the paper and helped to draft the manuscript. All authors read and approved the final manuscript.

## Pre-publication history

The pre-publication history for this paper can be accessed here:

http://www.biomedcentral.com/1471-2458/13/1/prepub

## Supplementary Material

Additional file 1 supplement. pdf — Supplemental information. Details of the studies identified by the literature review, along with the search strategy and selection criteria. Click here for file
