# Supplementary material for: A commentary on studies presenting projections of the future prevalence of dementia
Source: BMC Public Health. 2013 Jan 2;13:1. doi: 10.1186/1471-2458-13-1 (PMC3547813; doi:10.1186/1471-2458-13-1)
Supplement: Additional file 1 — supplement. pdf — Supplemental information. Details of the studies identified by the literature review, along with the search strategy and selection criteria. [file 1471-2458-13-1-S1.pdf]

Supplemental material: A commentary on studies presenting projections of the future prevalence of dementia

Literature search strategy and selection criteria

The aim of the literature review was to identify articles published in peer-reviewed journals presenting projections concerning the world as a whole or for individual continents. Relevant articles were identified from PubMed using the search '(alzheimer* or dementia) and (projection or forecast)'. Including only articles published in the last 10-years, written in English, and using adult humans aged 45+ resulted in 281 articles. Screening the title and abstract of these articles seventeen were selected for further inspection. Of these, two were excluded because they were either review articles or editorials, and a further two because they were not relevant (i.e. did not present projections). A further 8 were initially excluded as the projections concerned individual countries rather than continents. Of the remaining five studies, three presented projections for the entire world (1–3); four Europe (2–5); and two North America (2,3). For two studies estimates for certain regions could be combined to provide estimates for Latin America and the Caribbean, as well as Asia, Africa and Oceania (2,3).

Since the studies concerning North America included only the US and Canada in their estimates, we also included three projections concerning the US alone (6–8). This was because, by this definition, 90% of the North American population lives in the US.

Results

Details of these studies are given in Table 1 and the number of cases of dementia or AD projected in Figure 1. Of the studies three extrapolated prevalence estimates to existing population and projections (1,2,4) and five employed cell based macro

simulations based on discrete time multi-state models for the incidence of dementia or Alzheimer's disease (3,5–8). Two of the cell-based macro simulations additionally incorporated disease progression into the multi-state models (3,7).

Irrespective of method or region, the projected number of cases of dementia was predicted to rise considerably during the first half of this century. Due to the larger expected gains in life expectancy for developing countries, steeper increases in the estimated number of cases of dementia in the regions consisting of Latin America and the Caribbean, and Africa, Asia and Oceania compared to Europe and North America (2,3). For example, Ferri et al. (2) estimated that between 2000 and 2040 there would be a 229% increase in cases in Europe but a 534% increase in Latin America and the Caribbean. Moreover, the same study estimated that 59% of all cases of dementia would occur in Asia by 2050, compared to 48% in 2006.

References

1. Wimo A, Winblad B, Aguero-Torres H, von Strauss E. The magnitude of dementia occurrence in the world. Alzheimer disease and associated disorders. 2003 Jan;17(2):63–7.

2. Ferri CP, Prince M, Brayne C, Brodaty H, Fratiglioni L, Ganguli M, et al. Global prevalence of dementia: a Delphi consensus study. Lancet. 2005 Dec 17;366(9503):2112–7.

3. Brookmeyer R, Johnson E, Ziegler-Graham K, Arrighi HM. Forecasting the global burden of Alzheimer's disease. Alzheimer's & dementia : the journal of the Alzheimer's Association. 2007 Jul;3(3):186–91.

4. Wancata J, Musalek M, Alexandrowicz R, Krautgartner M. Number of dementia sufferers in Europe between the years 2000 and 2050. European psychiatry : the journal of the Association of European Psychiatrists. 2003 Oct;18(6):306–13.

5. Mura T, Dartigues J-F, Berr C. How many dementia cases in France and Europe? Alternative projections and scenarios 2010-2050. European journal of

neurology : the official journal of the European Federation of Neurological Societies. 2010 Feb;17(2):252–9.

6. Brookmeyer R, Gray S, Kawas C. Projections of Alzheimer's disease in the United States and the public health impact of delaying disease onset. American journal of public health. 1998 Sep;88(9):1337–42.

7. Sloane PD, Zimmerman S, Suchindran C, Reed P, Wang L, Boustani M, et al. The public health impact of Alzheimer's disease, 2000-2050: potential implication of treatment advances. Annual review of public health. 2002 Jan;23:213–31.

8. Hebert LE, Scherr PA, Bienias JL, Bennett DA, Evans DA. Alzheimer disease in the US population: prevalence estimates using the 2000 census. Archives of neurology. 2003 Aug;60(8):1119–22.

Table 1. Details of studies identified in the literature search

First author	Year	Condition	Population	Method	Period	Projection	Prevalence/Incidence Input	Mortality adjustment	Progression
Brookmeyer	1998	Alheimer's Disease	US	Macro simulation based on discrete time incidence model	1997>2047	US census bureau population projection	Incidence: Pooled estimates across 4 US studies, modelled as exponential function (Bachman 1993; Herbert 1995; Kokmen 1988; Shock, 1984)	RR = 1.44 (Evans, 1991)	na
Sloane	2002	Alheimer's Disease	US	Macro simulation based on discrete time incidence-progression model	2000>2050	US census bureau population projection	Prevalence: US General Accounting Office AD estimates, modelled as exponential function and calibrated to match GAO estimates	Assumed 10% higher for mild and 20% higher for mod/severe	Average 4yr progression (Doody 2001), assume yearly probability is .28
Wimo	2003	Dementia	World	Extraplotion of prevalence estimates	1950>2050	UN population projection	Prevalence: meta-analysis estimates (Fratiglioni & Rocca, 2001)	na	na
Hebert	2003	Alheimer's Disease	US	Macro simulation based on discrete time incidence model	2000>2050	US census bureau population projection	Incidence: estimates from a Chicago based poputlation study (Evans 2003)	RR = 1.44 (Evans, 1991)	na

Wancata	2003	Dementia; Alzheimer's Disease	Europe	Extraplotion of prevalence estimates	2000>2051	UN population projection	Prevalence: Pooled estimates across meta-analyses (Dem: Jorm 1987, Hofman 1991, Lobo 2000, Ritchie & Kildea 1995; AD: Rocca 1991, My & Kellar 2000, Lobo 2000)	na	na
Ferri	2005	Dementia	World; Europe; North America; Latin America & Carribean; Asia, Africa & Oceania	Extraplotion of prevalence estimates	2001>2040	UN population projection	Prevalence: Delphi consensus estimates	RR = 2.38 up to age 89; RR = 1.8 women age 90+; RR = 1.6 males age 90+ (EURODEM study: Jagger et al. 2000)	na
Brookmeyer	2007	Alheimer's Disease	World; Europe; North America; Latin America & Carribean; Asia, Africa & Oceania	Macro simulation based on discrete time incidence-progression model	2006>2050	UN population projection	Incidence: Estimates from meta-analysis (update Jorm & Jolley 1998)	Excess mortality risk 11% (calibrated against Brookmeyer, 2002)	Average 6-year progression (Neumann, 2001), assume yearly probability of .167
Mura	2010	Dementia	Europe	Macro simulation based on discrete time incidence model	2010>2050	Eurostat population projections	Incidence: Estimates from meta-analysis (Trataglioni 2000, EURODEM review)	Excess risk reduces with age: RR (65-74y) = 3.75; RR (75-84y) = 2.59; RR (85-y+) = 1.59 (Ostbye 1999)	na
